# Exploring the effects of risk-taking, exploitation, and exploration on divergent thinking under group dynamics

**DOI:** 10.3389/fpsyg.2022.1063525

**Published:** 2023-01-18

**Authors:** Tsutomu Harada

**Affiliations:** Graduate School of Business Administration, Kobe University, Kobe, Japan

**Keywords:** risk-taking, exploitation, exploration, Q learning, divergent thinking, loss aversion, computational approach

## Abstract

This study examined the effects of risk-taking and exploitation/exploration trade-off on divergent thinking in individuals, dyads, and triads. We adopted a simple Q-learning model to estimate risk attitudes, exploitation, and exploration parameters. The results showed that risk-taking, exploitation, and exploration did not affect divergent thinking in dyads. Instead, loss aversion was negatively related to divergent thinking. In contrast, risk attitudes and the inverse temperature as a ratio between exploitation and exploration were significant but with contrasting effects in individuals and triads. For individuals, risk-taking, exploitation and loss aversion played a critical role in divergent thinking. For triads, risk aversion and exploration were significantly related to divergent thinking. However, the results also indicated that balancing risk with exploitation/exploration and loss aversion is critical in enhancing divergent thinking in individuals and triads when learning coherence emerges. These results could be interpreted consistently with related literature such as the odd-vs. even-numbered group dynamics, knowledge diversity in group creativity, and representational change theory in insight problem-solving.

## Introduction

Understanding the determinants of creativity has been the subject of intensive research efforts. Creativity research involves several challenges related to the nature of solutions to creative problems, which are typically unpredictable (Metcalfe and Wiebe, 1987), difficult to report (Schooler et al., 1993), and solved in a distinct manner (Lavric et al., 2000). Thus, creativity requires new information that is often discrete and domain-specific, transcending informational boundaries but still providing some value. Creativity is defined as a combined manifestation of novelty and usefulness (Sternberg and Lubart, 1999; Jung et al., 2010). In particular, creativity has been, in many cases, identified with divergent thinking. Divergent thinking is defined as generating multiple solutions to an open-ended problem (Guilford, 1967). Thus, divergent thinking reflects that creativity is more likely to proceed unpredictably and abruptly (Baer, 1993; Runco and Acar, 2012).

As one of the candidates for the determinant of creativity, risk-taking attitudes have been extensively studied (Eisenman, 1987; Sternberg and Lubart, 1992; Feist, 1998; Dewett, 2007). Creative persons tend to favor challenging and risky situations (Albert, 1990; Perkins, 1990). Most related empirical studies reported that risk-taking was positively correlated to creativity (Eisenman, 1987; El-Murad and West, 2003; Dewett, 2007; Simmons and Ren, 2009; Tyagi et al., 2017; Harada, 2020, 2021a). However, some studies suggested that risk-taking did not account for divergent thinking (Shen et al., 2018). This difference related to risk-taking in creativity might be a result of cultural and atmospheric settings.

In addition to risk-taking, growing interest emerged in the effects of exploitation vs. exploration on creativity mainly in management literature (March, 1991; Tushman and O'Reilly, 1996; Birkinshaw and Gibson, 2004; Birkinshaw and Gupta, 2013; O'Reilly and Tushman, 2013; Turner et al., 2013). This concept was originally proposed in a reinforcement learning (RL) framework. In this context, exploitation refers to the optimization of current tasks under existing information and memory conditions, while exploration implies wider and sometimes random search and trials that do not coincide with the optimal solutions provided by exploitation (Sutton and Barto, 2018), indicating the trade-off between exploitation and exploration in the RL framework. Creativity requires both exploration and exploitation. In exploration, a wider search for a greater range of information is undertaken. In some cases, previously acquired knowledge must be unlearned so as not to get stuck with current knowledge, constraints or implicit assumptions, making it harder for them to ‘think outside the box’. At the same time, creativity also relies on exploitation because the efficiency of search in a much narrower space should take full advantage of existing information. Thus, both exploration and exploitation appear to be advantageous in creative thinking, although the relative weight of each depends on the phase of the creative thinking process.

According to representational change theory (Ohlsson, 1992; Knöblich et al., 1999), insight problem solving initially involves the construction of an erroneous problem space. Representational change takes place through the relaxation of constraints such as the abandonment of unnecessarily constraining assumptions. Risk-taking attitudes and exploration vis-à-vis exploitation provide a strong impetus for challenging the existing rules of the game to remove unnecessary constraints and create more appropriate problem spaces. Taken together, we hypothesize that divergent thinking is facilitated by positive risk-taking and exploration because new insights, as critical ingredients of divergent thinking, are considered to be a function of cognitive flexibility, which is enabled by the removal of underlying constraints.

While extensive studies exist regarding the determinants of individual divergent thinking, such as risk-taking (Eisenman, 1987; Sternberg and Lubart, 1992; Feist, 1998; Dewett, 2007) and exploitation/exploration trade-off (Harada, 2021a), less research has been done on the determinants of collective divergent thinking in terms of learning properties. Of course, there exists an explosion of studies on team innovation or creativity (Hülsheger et al., 2009) and on group creativity (Paulus, 2000; Nijstad and Paulus, 2003; Nijstad and Stroebe, 2006). However, these studies had difficulty in differentiating between social influence factors and cognitive ones because the cognitive processes derive from social interactive processes and the social influence processes are cognitively mediated (Paulus et al., 2012). As a result, these studies could not analyse the direct effects of cognitive and learning properties such as risk attitudes, exploitation and exploration at the group level on creativity or divergent thinking.

This study examined how risk attitudes and the exploitation/exploration trade-off influenced individual and group creativity in divergent thinking. Risk attitudes refer to risk-seeking and risk-averting behavior. Exploitation implies the optimal decision-making making use of current information. In contrast, exploration involves random choice. Thus, a trade-off exists between exploitation and exploration. For example, AlphaGo, a reinforcement learning program applied to the board game Go, consists of exploitation that selects the best moves based on the knowledge obtained through deep learning and exploration that selects the moves randomly to figure out new strategies for winning not discovered in past game records. The mixture of exploitation and exploration (or retaining their trade-off) facilitated AlphaGo’s learning. It defeated Ke Jie, the top-ranked player in the world, in 2017. Thus, the exploitation/exploration trade-off could facilitate divergent thinking because AlphaGo created several new strategies. Since risk attitudes and the exploitation/exploration trade-off were relevant to individual divergent thinking in a related study (Harada, 2020), conjecture that these learning properties could also exert some influence over group creativity is reasonable. To the best of our knowledge, this study was the first to examine these factors in group and individual creativity.

This study evaluated the creativity and learning performance of individuals, dyads, and triads using alternative use tests (AUT) and two-armed bandits (TAB), respectively. Given these measures, we examined the effects of risk attitudes on creativity across individuals, dyads, and triads. Learning properties such as risk-taking attitudes and the exploitation/exploration trade-off were estimated based on the Q learning framework applied to the observed behavior of individuals, dyads, and triads in the TAB tasks. In this Q learning model, exploitation implies the selection of choices yielding the highest Q values, whereas exploration involves a preference for other non-optimal choices. These factors can be represented *via* an inverse temperature scale using the softmax function, as described in the Methods section. On the scale, a higher (lower) value implies a greater (lower) emphasis on exploitation (exploration). This paper exploited this measure to examine the effects on divergent thinking performance in creative tasks. Risk-taking attitudes were also estimated in the Q learning framework where the prospect utility function (Tversky and Kahneman, 1986) was incorporated in this model. The prospect utility function assumes asymmetrical attitudes toward losses and gains. On the one hand, when faced with a risky choice leading to gains, agents prefer certain reward (say $10) to uncertain one even though its expected reward is equivalent to certain one (=$10), implying that agents are risk averse. On the other hand, when faced with a risky choice leading to losses, agents prefer uncertain loss to certain one because there exists a chance of no loss in the former case, implying that agents are risk taking (or risk seeking). Thus, in the prospect utility function, the utility function is concave over gains and convex over losses. By evaluating this utility function, we could reveal risk taking (or risk averting) attitudes. Consequently, by taking a computational approach to individual and group behaviors through a Q learning framework, related, but distinct concepts of risk-taking attitudes and the exploitation/exploration trade-off could be rigorously estimated.

Collective vis-à-vis individual decision-making in cognitive tasks has been extensively studied in related literature, though not directly in relation to creativity issues. Some argued that because individuals have incentives to withhold information strategically to gain or maintain an informational advantage, groups underperform individuals (Mitusch, 2006). However, most related literature has assumed that group members cooperate and share information voluntarily (Kerr and Tindale, 2004; Adamowicz et al., 2005; Tindale and Kluwe, 2015). This research has shown that groups outperform individuals in decision-making (Hinsz, 1990; Morgan and Tindale, 2002; Nijstad and Paulus, 2003; Kerr and Tindale, 2004; Maciejovsky and Budescu, 2007; Laughlin, 2011; Mellers et al., 2014). In particular, the signal detection model is one of the most rigorous approaches to collective decision-making (Sorkin et al., 2001; Bahrami et al., 2010, 2012a,b, 2013; Koriat, 2012; Bang et al., 2014; Mahmoodi et al., 2015; Pescetelli et al., 2016). Bahrami et al. (2010) showed that the result of interactive decision-making of two persons was better than a unilateral decision when the individuals shared a similar visual sensitivity and when they were presented with equal opportunities to communicate freely. However, if two individuals have different visual sensitivities, their performance was generally worse than that of a single decision-maker. The latter negative aspect of teams has also been pointed out in group dynamics literature, for example, group pressure (Bond and Smith, 1996; Coleman, 2004; Berns et al., 2005; Mori and Arai, 2010; Deuker et al., 2013; Yu and Sun, 2013), risky shift (Armstrong et al., 2004), social loafing (Latané et al., 1979), interpersonal competition (Hastie and Kameda, 2005), and group thinking (Janis, 1972; Packer, 2009), leading to group collective unintelligence. However, these studies did not directly refer to group creativity. Instead, they emphasized group decision-making in problem-solving or learning.

From the perspective of risk attitudes and exploration in collective decision-making, the studies on odd-vs. even-sized group dynamics (Hastie and Kameda, 2005; Menon and Phillips, 2011) directly relate to this study. This literature underscores that small groups are likely to break into two coalitions. When a group is even-sized, two equal-sized subgroups are likely to emerge, making it difficult to apply the majority rule. As a result, subgroup dynamics might lead to deadlock (Shears, 1967; Murnighan, 1978; Polzer et al., 2006; O'Leary and Mortensen, 2010; Harada, 2021b). In contrast, when a small group is odd-sized, minority and majority subgroups emerge. The majority influence provides a clear direction and group cohesion (Asch, 1951; Wittenbaum et al., 1996; Hastie and Kameda, 2005; Menon and Phillips, 2011; Harada, 2021b,c).

Consequently, learning coherence and incoherence take place with odd-numbered (individuals, triads) and even-numbered (dyads) groups, respectively (Harada, 2021c). Learning coherence is defined as a coherent use of learning strategies. Conversely, learning incoherence refers to their unintelligible or disorganized use. In dyads, in one moment, one member may make decision based on her learning preference, and in another moment, another member may take initiative in decision making. As a result, group learning strategy is likely to become incoherent over time. In contrast, because majority rule can be applied to triads, majority subgroups may make decisions based on their own learning strategies; thus, group learning strategies may be more coherent. Issues of learning coherence and incoherence could emerge in collective decision-making. Their emergence, in turn, affects risk-taking and exploration’s roles in divergent thinking. For odd-numbered groups, risk-taking and exploration is required to break consensus from the majority rule. However, with even-numbered groups, conflict, rather than consensus, is more likely to emerge. This conflict challenges current constraints and implicit assumptions and increases exposure to diverse perspectives, which should increase creativity (Amabile, 1996). Thus, even-numbered groups do not require risk-taking and exploration to facilitate divergent thinking. Instead, they need to integrate diverse information and reconcile differing perspectives, which may stimulate creative thinking (Knippenberg et al., 2005). Hence, risk-taking might not be necessary for divergent group thinking. Instead, greater risk-aversion or exploitation might be necessary.

Another straightforward effect of encouraging divergent thinking in decision-making is group diversity. A larger group usually leads to increased diversity of perspectives, learning strategies, and ideas. In addition, creativity is enhanced by groups diverse in experience and expertise (Paulus et al., 2012). Nevertheless, systematic empirical studies on diversity’s effects on group creativity show somewhat mixed results (Mannix and Neale, 2005; Knippenberg and Schippers, 2007). Moreover, related meta-analyses (Bowers et al., 2000; Webber and Donahue, 2001) found no consistent evidence that group diversity improved performance. These inconsistent results suggest that the diversity’s effects depend on the type at issue. On the one hand, diversity associated with personal and demographic characteristics such as age, gender, and race could inhibit social interaction, communication, and teamwork. This inhibition leads to lower group performance (Mannix and Neale, 2005). Since these differences are not necessarily related to experience and expertise required for creative tasks, diversity in surface-level characteristics could adversely affect group creativity. On the other hand, knowledge diversity could enhance group creativity. It increases the knowledge base leading to the generation of more ideas (Knippenberg and Schippers, 2007). Hence, while demographic diversity is inversely related to creativity, knowledge diversity affects it positively (Hülsheger et al., 2009). If more group size increases knowledge diversity, triads should outperform dyads, and dyads outperform individuals. As a result, groups characterized by more knowledge diversity do not necessarily require risk-taking. On the contrary, integrating diverse ideas to generate new, useful ones might necessitate more risk aversion and exploitation. Thus, under collective decision-making, the role of risk-taking and exploration in divergent thinking could have mixed effects. On the one hand, odd-numbered groups require risk-taking and exploration for divergent thinking. On the other hand, risk aversion and exploitation, rather than risk-taking and exploration, is required for divergent thinking as group size increases.

Consequently, it is predicted that individuals require risk-taking and exploration. In contrast, triads require risk-taking behavior and exploration (for challenging current contexts and constraints) and risk-aversion and exploration (for integrating diverse ideas). As described later, risk attitudes over gains and losses were separately estimated in the prospect utility function. Agents could show different risk attitudes related to gains and losses. For example, they might be risk-taking over losses and risk-averting over gains. In contrast, dyads require neither risk-taking nor exploration in divergent thinking. Instead, creativity may need risk-aversion and exploitation. This study examined these hypotheses.

## Materials and methods

### Participants

A sample of 431 healthy undergraduate students (171 females) at X University with the age range of 18–20 years (mean = 18.92, SD = 0.77, median = 19) participated in experiments for course credit. All participants and their academic advisers signed informed consent before the experiment, approved by the local Ethics Committee at the Graduate School of Business Administration, X University. Out of 431, 78 participants participated in the experiments as individuals, 170 formed 85 dyadic groups, and 183 participants brought about 61 triadic groups. All participants were assigned to work as individuals, dyadic or triadic groups without duplication.

### Experiments

In Test 1, participants undertook the AUT. In test 2, the same dyadic or triadic groups undertook the two-armed bandit test (TAB), performed with PsyToolKit (Stoet, 2010, 2017). About a half of them worked on AUT first and TAB next, and the remaining half took these tests in the opposite order to eliminate order effects.

### Alternative use test

**Figure 1:**
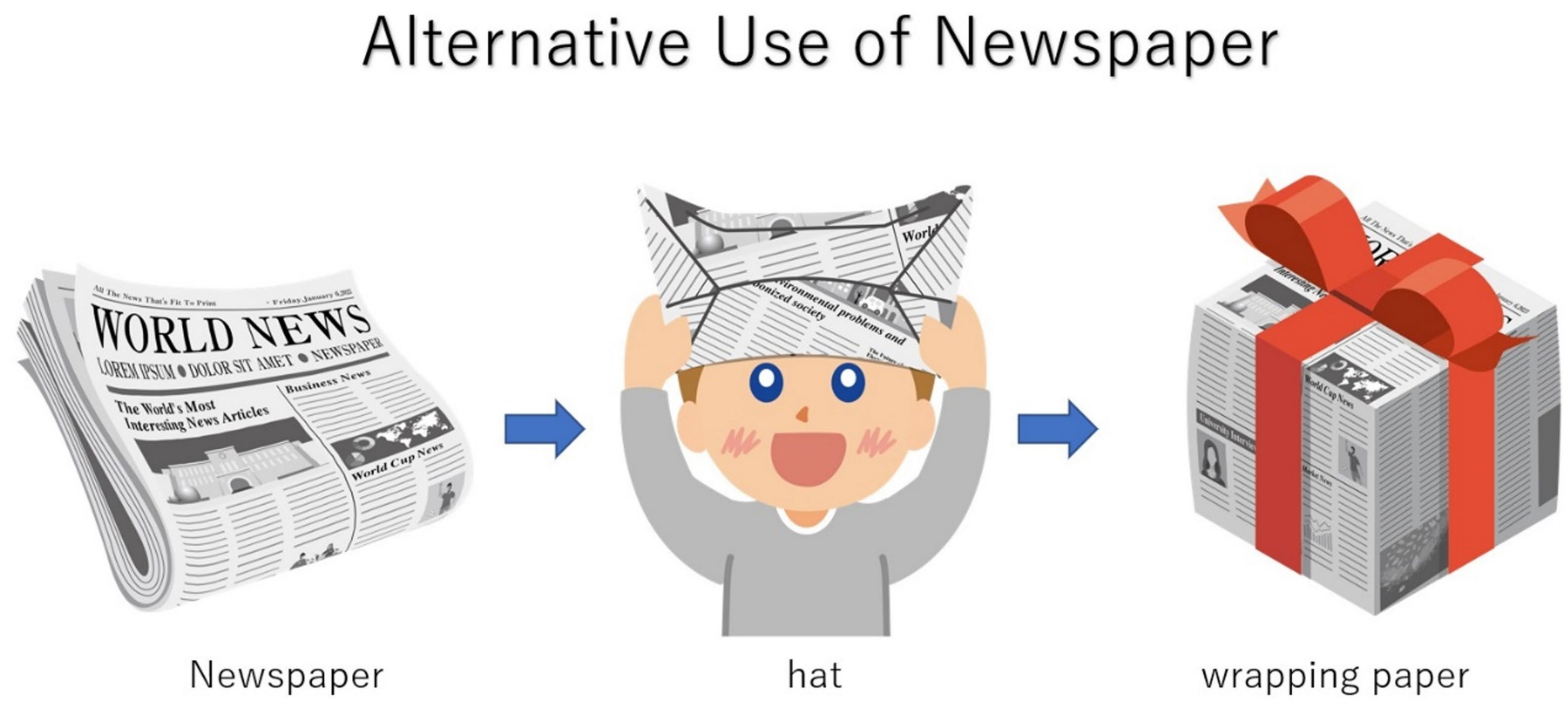
In the AUT, participants were asked to consider some common objects. Each object had a common use, which was stated in the answer sheets. They were asked to list other possible uses of the object or the part of the object. In this example, an object is a newspaper (used for reading). The alternative uses were listed as a hat and wrapping paper.

Divergent thinking is the ability to produce new approaches and original ideas by forming unexpected combinations from available information and applying semantic flexibility and fluency of association, ideation, and transformation (Guilford, 1967). The current study measured divergent thinking ability with the AUT, a timed laboratory test corresponding to the Torrance Test of Creative Thinking. The AUT requires test-takers to generate as many alternative ways of using three objects (shoes, buttons, and keys to lock and open a door) as possible (up to 10) within 8 minutes. While several alternative tests exist for divergent thinking, this study adopted the AUT as this test is a reliable indicator of creative potential (Runco and Acar, 2012) and has been extensively used by many related studies (Figure 1).

In the current study, the AUT was measured in accordance with the instructor manual of the S-A creativity test (Society for Creative Minds, 1969), a timed laboratory test corresponding to the measures used in the Torrance Tests of Creative Thinking. The AUT measures divergent thinking in terms of (a) fluency, (b) flexibility, (c) originality, and (d) elaboration. These scores are calculated based on a criteria table consisting of two dimensions. One dimension indicates expected idea categories into which responses may fall. Each idea category has an originality weight of either 0 or 1. If a category indicates a statistical rarity of response (i.e., it is unlikely that a respondent provides answers that cover this category), 1 point is assigned. Otherwise, the originality weighting is 0 points. Another dimension indicates the degree of concreteness of the responses, consisting of “ambiguous response” (0 points), “either objective or method described” (1 point), and “both objective and method described” (2 points). Each response was assigned using this two-dimensional table or nearly equivalent judgment.

Fluency is measured by the number of relevant responses to the questions. This skill represents the ability to produce and consider many alternatives. Flexibility is the ability to produce responses from a broad perspective. It is measured by the total number of different idea category types in which relevant responses are assigned on the criteria table. Originality is the ability to produce novel ideas. Its scoring is the sum of different idea category types with the originality weighting of a category covered by relevant responses in the criteria table. For example, suppose α,β, and γ responses respectively, cover category A with the originality weighting of one, category B with the originality weighting of zero, and L different category types (not including category A), each of which has an originality weighting of one. The originality score thus amounts to 1 + L. Finally, elaboration is the production of ideas in detail. It is measured by the sum of the points measured by the degree of concreteness covered by relevant responses on the criteria table. For example, if α,β, and γ responses represent “ambiguous response” (0 points), “either objective or method described” (1 point), and “both objective and method described” (2 points), respectively, the elaboration score amounts to β+2γ. This test also provides a total score for divergent thinking used in this paper.

Two graduate students (1 female, mean age = 35) majoring in organizational psychology were recruited to evaluate the scores of fluency, flexibility, originality, and elaboration for 224 responses with payment of JPY 100,000 (approximately USD 850) for each. They independently evaluated these scores according to the above procedures. The interrater reliability estimates were ICC (2,2) = 0.87 [*F*(223,223) = 9.9, *p* = 1.3e-67]. The rounded average scores were used for subsequent analysis.

### Q learning model

**Figure 2:**
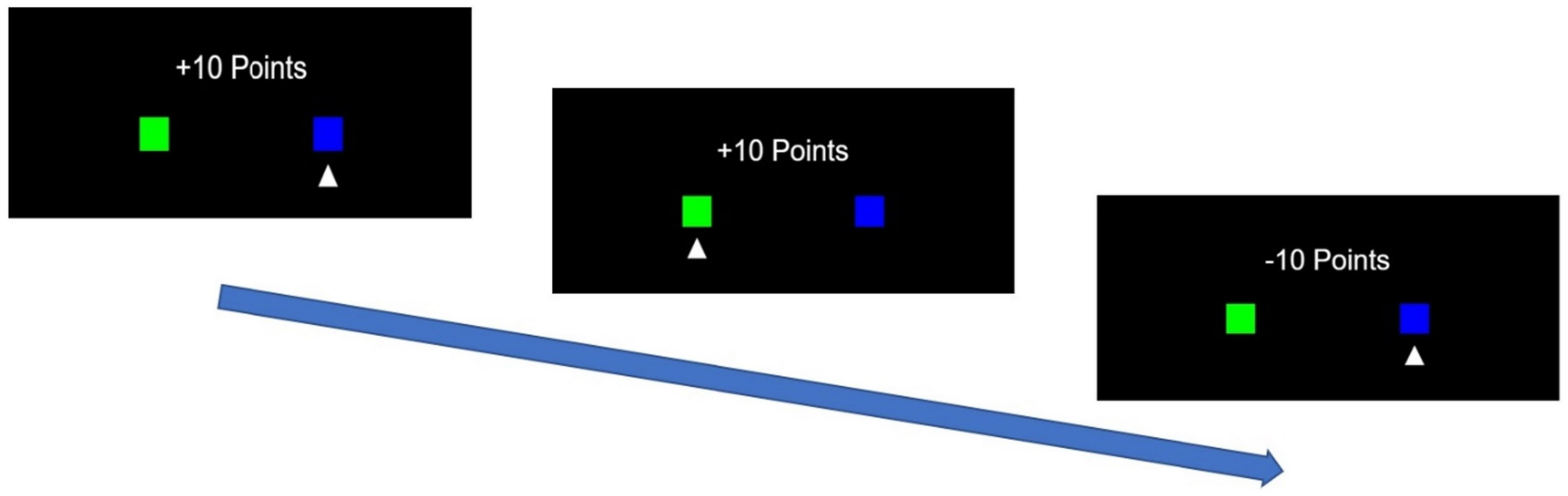
Example of a trial in the two-armed bandits in which the participant chose the right box first, then the left, and finally the right, with rewards of 10 points, 10 points, and −10 points, respectively.

A simple Q-learning reinforcement learning algorithm (Watkins and Dayan, 1992) incorporating the prospect utility function (Tversky and Kahneman, 1986), proposed by Harada (2020), was adopted to measure learning properties and risk attitudes of individuals, dyadic and triadic groups. In a TAB problem, participants were asked to choose either a right or left box on the screen. After selecting a box, a reward appeared on the screen, either 10 or-10. Participants were instructed to maximize the total rewards through a series of 100 choices. One of the boxes was programmed to give 10 points with a higher probability (70%), and the corresponding probability of the other box was set at 30%. It was expected that some participants quickly learn which of the two boxes would yield higher rewards and keep selecting that box in the subsequent choices. To avoid this convergence, we switched these probabilities twice over 100 choices. For the first 30 choices, the right and left boxes were set to have a respective 70 and 30% probability of yielding 10 points. From the 31st to the 70th choice, the probabilities switched between the right and left boxes. For the last 30 choices, these returned to exact probabilities during the first 30 choices (Figure 2).

To measure the risk-taking attitude observed in decision-making in the TAB, we used a variant of the Q learning model in the RL framework (Sutton and Barto, 2018). At each trial t, the action value $Q_{i}\left(t\right)$ of the chosen option (box) i is updated through the following rule:


(1) $$Q_{i}\left(t+1\right)=\{\;\begin{array}{c} Q_{i}\left(t\right)+\alpha^{+}\delta \left(t\right)+\phi \;\mathrm{if}\;\delta \left(t\right)\geq 0,\\ \;Q_{i}\left(t\right)+\alpha^{-}\delta \left(t\right)+\phi \;\mathrm{if}\;\delta \left(t\right)<0\text{,} \end{array}$$

with


(2) $$\delta \left(t\right)=U\left(R_{i}\left(t\right)\right)-Q_{i}\left(t\right)\text{,}$$

(3) $$U\left(R_{i}\left(t\right)\right)=\{\begin{array}{c} R_{i}{\left(t\right)}^{\mu }\;\mathrm{if}\;R_{i}\left(t\right)>0,\\ -\lambda {\left(-R_{i}\left(t\right)\right)}^{\nu }\;\mathrm{if}\;R_{i}\left(t\right)<0\text{,} \end{array}$$

where $R_{i}\left(t\right)$is the reward when option i is selected at trial t, which is either 10 or −10. The updating Equation 1 differs from the standard Q model in that the learning rates $\alpha^{\pm }$are assumed to be asymmetric between positive and negative $\delta \left(t\right)$ (reward prediction errors). Cazé and van der Meer (2013) showed that even in simple, static bandit tasks, agents with differential learning rates can outperform unbiased agents. If $\alpha^{+}-\alpha^{-}$ is positive (negative), the positivity (negativity) bias exists, implying that agents are more responsive to gains (losses) rather than losses (gains) in$\delta \left(t\right)$. We introduced differential learning rates, although learning biases were not our primary concern, because the prospect utility function required different learning rates for gains and losses. λ evaluates losses relative to gains, usually referred to as the loss aversion. A higher λ implies agents want to avoid losses. Note that λ measures the sensitivity to negative rewards, while risk attitudes evaluate the sensitivity to changes in rewards.

$\delta \left(t\right)$ refers to the reward prediction errors, measuring the gap between the actual utility of gaining $R_{i}\left(t\right)$ and the current value estimate $Q_{i}\left(t\right)$. If this value is positive (negative), $Q_{i}\left(t+1\right)$ is updated to increase (decrease) from the previous $Q_{i}\left(t\right)$. The learning rates adjust this change in updating. Learning rates close to 1 indicate fast adaptation, and learning rates closer to 0 imply slow adaptation. In the default setting, the initial action values were set to zero so that $Q_{i}\left(1\right)=0$ for i=1,2. In some cases, participants might have tendency to select the same choice over time. This autocorrelation of choices could bias the magnitudes of learning rates $\alpha^{\pm }$ (Katahira, 2018). To correct this bias, ϕ was added in Equation 1. $U\left(R_{i}\left(t\right)\right)$ adopts the form of the prospect utility function (Tversky and Kahneman, 1986) to measure risk attitude whose functional forms depend on the sign of $R_{i}\left(t\right)$. μ and νin Equation 3 measure the degrees of risk aversion and risk-seeking, respectively. Risk-seeking (aversion) is related to lower (higher) μ and higher (lower) ν in this specification.

For the unselected option, $j\;\left(i\neq j\right)$, $Q_{i}\left(t+1\right)$ updates without any changes.


(4) $$Q_{j}\left(t+1\right)=Q_{j}\left(t\right)\text{.}$$

Denote that the chosen action at trial t by$a\left(t\right)\in \left\{1,2\right\}$. The probability of choosing an option is assumed to follow the softmax decision rule: T


(5) $$P\left(a\left(t\right)=i\right)=\frac{\exp\left(\beta Q_{i}\left(t\right)\right)}{\sum_{j=1}^{2}\exp\left(\beta Q_{j}\left(t\right)\right)}\text{,}$$

where $P\left(a\left(t\right)=i\right)$ is the probability of choosing the action $a\left(t\right)=i$ at trial t andβ is the inverse temperature, measuring the sensitivity of a participant’s choice to the difference in action value estimates.

These parameters were estimated by maximizing the posteriori (MAP) objective function:


(6) $$\hat{\theta }=\mathrm{argmax}\;p\left(D_{s}|\theta_{s}\right)p\left(\theta_{s}\right)\text{,}$$

where$p\left(D_{s}|\theta_{s}\right)$is the likelihood of data $D_{s}$ for subject s conditional on parameters $\theta_{s}=\left\{{\alpha^{\pm }}^{S},\mu^{S},\nu^{S},\lambda^{S},\phi^{S},\beta^{S}\right\}$, and $p\left(\theta_{s}\right)$is the prior probability of $\theta_{s}$. Some of these parameters were assumed to be bounded. Since $\alpha^{\pm }$ is bounded between 0 and 1, and μ,ν,λ, and βtake non-negative values, their priors of $\alpha^{\pm }$were assumed to follow beta distributions, and μ,ν,λ, and β to follow gamma distributions.

### Measures

This study examined the differential effects of risk-taking on divergent thinking across individuals, dyads, and triads. In addition, divergent thinking scores, calculated as the sum of (a) fluency, (b) flexibility, (c) originality, and (d) elaboration scores in AUT, were used as dependent variables. The risk attitudes toward gains and losses were, respectively, measured by *μ* and ν in Equation 3. Loss aversion was measured by λ.

We were also interested in examining the effect of the inverse temperature β because it represents levels of exploitation and exploration. Exploitation indicates the optimization under existing information and memory conditions, implying the selection of the option with the maximum Q value. Exploration refers to random search and trials, regardless of Q values. Exploitation alone does not necessarily lead to optimization because it deters from gaining information from unknown choices. Thus, exploration is also required to maximize the total rewards. The trade-off between exploitation and exploration in the RL framework (Sutton and Barto, 2018) is reflected in the inverse temperature β. A higher β value implies that the participants tend to select the boxes with the largest Q, leading to exploitation. Conversely, as β gets close to zero, the participants are more likely to choose boxes randomly because the weight of the Q value in Equation 3 decreases. At β=0, each choice has the same probability of being selected by the participants, and the Q values have no relevance to this probability. Hence, the inverse temperature β reflects the relative importance of exploitation vs. exploration. When we describe exploitation (exploration) as positively significant in the subsequent analysis, it means that β is significantly positive (negative).

Learning rates $\alpha^{\pm }$ and the autocorrelation measure of ϕ in Equation (1) were explanatory variables because they could also affect divergent thinking. Finally, the reward total in the TAB that could measure learning efficiency was adopted. Participants could achieve higher rewards if they correctly predicted the box with a higher probability of gains through trial and error.

Tables 1A-D, show the descriptive statistics of the pooled sample and its subsamples of individuals, dyads, and triads. It revealed that no significant correlation exists for most of the pairs of variables.

**Table 1A:** Descriptive statistics (pooled sample).

	Mean	SD	1	2	3	4	5	6	7	8
1. Divergent Thinking	55.88	19.36	—							
2. TAB performance	2.9	8.81	0.18***	—						
3. *β* (Inverse temparature)	2.53	2.12	−0.02	0.06	—					
4. *μ* (risk aversion in gains)	0.54	0.29	0.05	0.03	0.08	—				
5. *ν* (risk-seeking in losses)	0.49	0.29	−0.04	0.06	0.06	0.00	—			
6. *α*+ (learning late)	0.47	0.25	0.06	0.01	−0.18***	−0.03	0.02	—		
7. *α*− (learning late)	0.48	0.27	−0.04	−0.08	0.08	−0.06	−0.10	−0.01	—	
8. *λ* (loss aversion)	0.5	0.31	−0.04	0.05	0.05	−0.04	0.04	−0.01	0.07	—
9. *Φ* (autocorrelation control)	−5.61	47.48	0.02	0.02	0.08	−0.09	0.04	−0.20***	0.00	−0.04

*N* = 224. ***p < 0.01.

**Table 1B:** Descriptive statistics (individuals).

	Mean	SD	1	2	3	4	5	6	7	8
1. Divergent Thinking	53.79	20.76	—							
2. TAB performance	8.7	9.54	0.07	—						
3. *β* (Inverse temparature)	2.38	1.96	0.09	0.02	—					
4. *μ* (risk aversion in gains)	0.55	0.29	−0.10	−0.06	0.11	—				
5. *ν* (risk-seeking in losses)	0.51	0.29	−0.14	−0.03	0.08	−0.12	—			
6. *α*+ (learning late)	0.53	0.23	0.00	0.01	0.05	−0.08	0.06	—		
7. *α*− (learning late)	0.49	0.3	0.06	0.02	0.27	0.05	−0.09	0.07	—	
8. *λ* (loss aversion)	0.57	0.3	0.08	−0.07	0.10	0.11	0.15	0.01	0.13	—
9. *Φ* (autocorrelation control)	−8.08	47.74	0.00	−0.10	0.08	−0.10	−0.07	−0.29***	−0.01	−0.02

*N* = 78. ***p < 0.01.

**Table 1C:** Descriptive statistics (dyads).

	Mean	SD	1	2	3	4	5	6	7	8
1. Divergent Thinking	49.26	15.49	—							
2. TAB performance	−15.1	7.31	0.15	—						
3. *β* (Inverse temparature)	2.52	2.05	−0.13	0.13	—					
4. *μ* (risk aversion in gains)	0.52	0.3	0.07	0.21**	0.14	—				
5. *ν* (risk-seeking in losses)	0.45	0.29	0.01	0.12	0.07	−0.06	—			
6. *α*+ (learning late)	0.46	0.26	0.24**	0.12	−0.33***	−0.12	0.07	—		
7. *α*− (learning late)	0.48	0.24	−0.10	−0.17*	−0.07	−0.23**	−0.14	−0.08	—	
8. *λ* (loss aversion)	0.47	0.31	−0.19*	0.07	0.24**	−0.11	0.06	−0.20*	0.12	—
9. *Φ* (autocorrelation control)	−7.61	62.04	−0.03	0.12	0.12	−0.13	0.12	−0.19*	−0.01	−0.04

*N* = 85. *p < 0.10, **p < 0.05, ***p < 0.01.

**Table 1D:** Descriptive statistics (triads).

	Mean	SD	1	2	3	4	5	6	7	8
1. Divergent Thinking	67.77	17.11	—							
2. TAB performance	20.7	9.4	0.22*	—						
3. *β* (Inverse temparature)	2.74	2.4	−0.11	0.02	—					
4. *μ* (risk aversion in gains)	0.56	0.3	0.21	−0.07	−0.02	—				
5. *ν* (risk-seeking in losses)	0.51	0.29	−0.09	0.07	0.03	0.19	—			
6. *α*+ (learning late)	0.42	0.23	0.07	−0.11	−0.20	0.16	−0.13	—		
7. *α*− (learning late)	0.5	0.28	−0.21	−0.17	0.00	−0.03	−0.08	−0.02	—	
8. *λ* (loss aversion)	0.47	0.32	−0.01	0.16	−0.20	−0.12	−0.14	0.14	−0.08	—
9. *Φ* (autocorrelation control)	0.34	4.19	0.00	−0.26**	−0.06	−0.06	0.25*	−0.07	0.14	−0.23

*N* = 61. *p < 0.10, **p < 0.05.

## Results

### Comparison of performance

**Figure 3:**
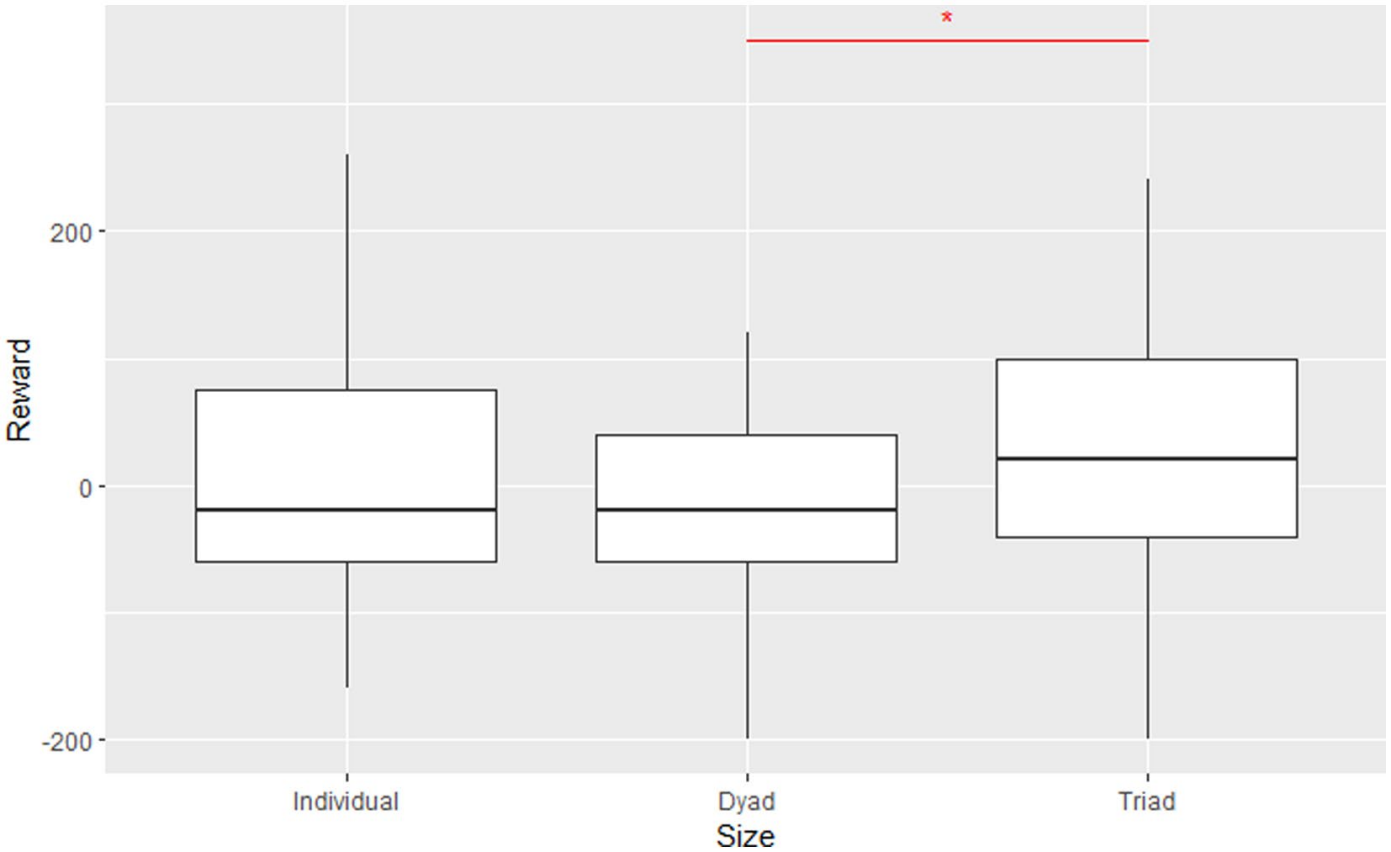
Comparison of average performance of individuals, dyads, and triads. Error bars represent standard errors of means. The Kruskal–Wallis test was applied. *p < 0.1.

Before examining the effects of risk attitudes and other learning properties on divergent thinking, we compared the relative performance of individuals, dyads, and triads regarding TAB and divergent thinking. According to the odd-vs. even-numbered group effects, the performance (the sum of rewards in the TAB) of individuals and triads should be higher than that of dyads because learning coherence is more likely to be achieved in the odd-numbered groups (Harada, 2021c). Indeed, U-shaped relationship was observed across individuals, dyads, and triads, suggesting that the odd-numbered individuals and triads outperformed dyads. The Kruskal-Wallis test was applied to examine these differences, since the data rejected the homogeneity of variance or normality by the Bartlett or the Shapiro–Wilk tests. The average performance for individuals, dyads, and triads was 8.7, −15.1, and 20.7, respectively. The Kruskal-Wallis test revealed significant group size effects on performance ($\chi_{2}^{2}=$ 5.06, *p* = 0.07). Then, the pairwise Wilcoxon Rank-Sum Test with Bonferroni adjustment presented significant differences in performance existing between dyads and triads (*p* = 0.06). Still, no significant differences were observed between individuals and dyads and between individuals and triads. This result suggested that “three heads are better than two.” (In other words, adding one member to a dyad improves performance.) Although the average performance of individuals was higher than that of dyads, this difference was not significant. This result is consistent with the previous studies (Menon and Phillips, 2011; Harada, 2021b) regarding dyads and triads. Thus, it could be inferred that more learning coherence (in terms of learning strategies over time) is likely to be observed in triads (Harada, 2021c). That is, triadic groups tend to adopt consistent learning strategies. For example, they might always select a box with higher cumulative scores (higher Q value) at the time. Alternatively, they might select a box randomly, regardless of past scores (Figure 3).

**Figure 4:**
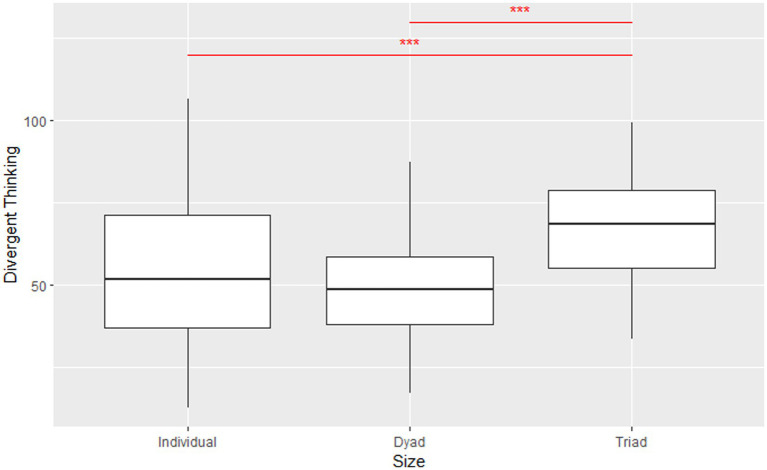
Comparison of AUT scores of individuals, dyads, and triads. Error bars represent standard errors of means. The Kruskal–Wallis test was applied. ***p < 0.1.

However, learning coherence does not necessarily lead to greater divergent thinking because diversity resulting from learning incoherence could contribute to higher scores in divergent thinking. The average performance for individuals, dyads, and triads was 53.54, 49.02, and 67.56, respectively. Once again, the Kruskal-Wallis test revealed the significant group size effects on performance ($\chi_{2}^{2}=$7.15, *p* = 0.03). The pairwise Wilcoxon Rank-Sum Test with Bonferroni adjustment presented significant differences in performance existing between individuals and triads (*p* = 1.9e-4) and between dyads and triads (*p* = 1.5e-8.). Thus, the performance was highest for triads, suggesting that three thinkers outperformed one or two (Figure 4).

### Effects on divergent thinking

To test our hypotheses, we conducted a regression analysis to examine the effects of risk attitudes and learning properties on divergent thinking for individuals, dyads, and triads. The dependent variables were total scores of divergent thinking and explanatory variables consisting of risk attitudes, inverse temperature, learning rates, constant terms accounting for autocorrelation of choices (ϕ), and the total sum of rewards in the TAB. Table 2 shows the results.

**Table 2:** Poisson regression results (SE in parentheses).

Variables	AUT scores		
	Individuals (1)	Dyads (2)	Triads (3)
Constant terms	61.27***	48.98***	75.38***
	(3.68)	(3.94)	(4.74)
*β* (inverse temparature)	1.13**	−0.41	−0.83*
	(0.46)	(0.40)	(0.45)
*μ* (risk aversion in gains)	−10.47***	2.59	16.02***
	(3.01)	(2.85)	(3.78)
*ν* (risk-seeking in losses)	−15.02***	−0.51	−13.66***
	(3.00)	(2.73)	(3.88)
*α*+ (learning rate)	−3.50	11.63***	0.81
	(3.89)	(3.32)	(4.79)
*α*− (learning rate)	0.35	−3.37	−14.20***
	(2.90)	(3.37)	(3.97)
*λ* (loss aversion)	8.26***	−6.73**	−4.08
	(2.91)	(2.68)	(3.56)
*Φ* (autocorrelation)	−0.02	0.00	0.62**
	(0.02)	(0.01)	(0.29)
TAB performance	0.16*	0.24**	0.52***
	(0.09)	(0.11)	(0.12)
AIC	1,064.60	878.2	601.89

*N* = 78 for individuals, *N* = 85 for dyads, and *N* = 61 for triads. The dependent variables are AUT scores. *p < 0.10, **p < 0.05, ***p < 0.01.

The effects of risk attitudes on divergent thinking were significant in individuals and triads, but not in dyads. The latter result partly supports our hypothesis. Both high divergent thinking individuals and triads were likely to avoid risk in the face of losses (negative coefficient on ν). However, as for positive rewards, high divergent thinking individuals were risk-seeking (negative coefficient on *μ*. In contrast, risk aversion characterized such triads (positive coefficient on *μ*. Thus, individuals were risk-seeking for gains and risk averting for losses, but triads were risk averting for both gains and losses. This finding also supports part of our hypothesis that individuals require risk-taking. Although we predicted that triads require both risk-taking and risk aversion, the results indicated that high divergent thinking triads required risk aversion alone for both gains and losses. This finding suggests that larger group size and resulting knowledge diversity (Hülsheger et al., 2009) in triads lead to more significant divergent thinking. Hence, instead of risk-seeking, triads necessitate risk aversion to convert diverse ideas into consistent ones.

Risk averting behaviors in triads were also complemented by the negative effect of the inverse temperature on divergent thinking. Since the inverse temperature β reflected the exploitation vs. exploration ratio, its negative impact implies that high divergent thinking triads were more related to exploration than exploitation. In contrast, high divergent thinking individuals were complemented by high inverse temperature, implying more exploitation. It follows that individuals achieved high divergent thinking scores through risk-seeking for gains, risk averting for losses, and exploitation. In contrast, high divergent thinking triads were associated with risk aversion and exploration. The results suggest that exploitation/exploration and risk attitudes were complementary. If more exploitation is pursued during the task, divergent thinking requires more risk-taking behavior. Conversely, if more exploration is pursued, divergent thinking should be complemented by risk aversion. Finally, high divergent thinking triads were related to risk aversion in the face of gains and losses, implying that knowledge diversity arising from a larger group size necessitates more risk aversion than dyads and individuals.

Regarding other learning properties, ϕ positively affected divergent thinking in triads, suggesting that a tendency to select the same box over time facilitates divergent thinking. This result seems somewhat counter-intuitive. If ϕ implies the adherence to past behavior, it should impede divergent thinking by definition. However, this adherence does not stifle divergence at all in triads. Higher ϕ might be more related to learning coherence in triads. When triadic groups find out which box is more likely to generate positive rewards, they continue to select the same box over time until the probability of giving higher rewards shifts. A possible interpretation is that higher ϕ leads to divergent thinking because they have high learning abilities.

The learning rates $\alpha^{+}$ and $\alpha^{-}$ showed contrasting effects between dyads and triads. $\alpha^{+}$ was positively associated with divergent thinking in dyads, while $\alpha^{-}$ was negatively related to divergent thinking in triads. Although these results seem mutually inconsistent, they share the same property of positive learning attitudes from the results. Positive $\alpha^{+}$ implies high sensitivity to learning from gains positively, whereas negative $\alpha^{-}$ is conducive to learning from losses positively (increasing, instead of reducing, the corresponding Q value), both of which promote learning positively from the results (increasing the corresponding Q value). High divergent thinking seems to be related to these positive learning from the results in dyads and triads. The loss aversion λ was related to divergent thinking positively in individuals, but negatively in dyads. Creative individuals want to avoid losses, but creative dyadic groups prefer losses. This might also reflect learning coherence of individuals and learning incoherence of dyads. Finally, the total sum of rewards for the TAB were all positively significant in individuals, dyads, and triads. The TAB scores measured learning efficiency and coherence. TAB scores critically depend on detecting a box with a higher probability of positive reward and realizing a probability change throughout 100 trials. Learning efficiency should be high, and learning strategy should be coherent over time to achieve this result.

## Discussion

This study examined the effects of risk attitudes and learning properties such as the inverse temperature on divergent thinking. It was predicted that (a) individuals require risk-taking, but (b) triads require both risk-taking and risk aversion, whereas (c) dyads need neither risk-taking nor risk aversion in divergent thinking. The results were consistent with (a) and (c) but did not fully support (b). That is, high divergent thinking triads were related to risk aversion in the face of both gains and losses, and risk-taking did not exert a significant effect on divergent thinking. Therefore, the results should be interpreted in terms of risk attitudes and the inverse temperature (exploitation vs. exploration ratio) rather than focusing on risk attitudes alone. As for individuals, challenges to current constraints and implicit assumptions were enabled by risk-taking in the face of gains, while more convergent thinking was born out by exploitation and risk aversion in the face of losses. As for triads, going beyond current contexts and constraints (in the case of AUT, thinking about alternatives to an object’s current primary use) was enabled by a group size of three and its attendant knowledge diversity, compared to dyads and individuals. In contrast, more convergent thinking was related to risk aversion in the face of both gains and losses. Both convergent thinking and risk aversion tend to generate convergence to the box selected over time. Thus, as far as the odd-numbered groups, including individuals, some balance could be required for divergent thinking between risk-taking (aversion) and exploitation (exploration). For dyads, learning incoherence (changing learning strategies over time) challenged existing contexts, such as cumulative scores of boxes in the TAB and primary use of an object in the AUT, without resorting to risk-taking and exploration.

However, the results regarding the effects of loss aversion between individuals and dyads differed. Divergent thinking was related to loss aversion positively in individuals and negatively in dyads. This suggests that the effects of loss aversion should be interpreted with reference to risk attitudes and the inverse temperature. In individuals, risk-taking behavior is complemented by inverse temperature (exploitation) and loss aversion. In dyads, loss aversion is reinforced by learning incoherence.

The study results are subject to several limitations. First, the robustness of the results should be further examined in future studies as cultural and generational backgrounds could influence them. If the same experiments are conducted in different settings, the results might differ from the current study. Second, the sample in this study was limited to a very narrow age range: 18–20 years. Notably, several studies reported that divergent thinking tends to change with age (Foos and Boone, 2008; Massimiliano, 2015; Fusi et al., 2021). However, this study did not account for these dynamic changes in divergent thinking. Therefore, these possible trends should be verified in future studies comprising a sample with a wide age range and education status. Third, the results critically depend on the creativity tasks (AUT) and learning tasks (TAB). Although it is common to adopt the AUT as a measure for divergent thinking and creativity in related studies, performance in the AUT alone is not sufficient for creativity as it consists of convergent and divergent thinking. The AUT does not require much convergent thinking because problem-solving activities are not included to devise some solutions. Instead, listing alternative uses of words are needed. Yet, for creativity to compete in the real world, proposed alternative uses must be transformed into concrete objects reflecting these ideas. Materializing and realizing new ideas relies more heavily on convergent thinking. Thus, creativity in the real world requires both divergent and convergent thinking. The learning properties measured through the TAB are also task-specific, results should be carefully interpreted.

Nevertheless, the results in this study are consistent with some of the previous studies empirically and theoretically. First, the role of risk-taking in divergent thinking in individuals was also reported in the related studies (Eisenman, 1987; El-Murad and West, 2003; Dewett, 2007; Simmons and Ren, 2009; Tyagi et al., 2017; Harada, 2020, 2021a), though subtle differences exist. Moreover, the present study differs in that dyads and triads were examined. The results indicated that depending on the group size, the role of risk-taking differed across individuals, dyads, and triads, which is new to related literature. One of the contributions of this study is that it examined creativity under collective decision-making. Further investigation is obviously required regarding creativity under collective decision-making and group dynamics. Second, from a theoretical perspective, the results underscored the importance of balancing both boldness (risk-taking, exploration, learning incoherence) and prudence (risk-aversion, exploitation, loss aversion) in individuals and triads. In dyads, low loss aversion could be a result of learning incoherence. Hence, these balances are effective under learning coherence in individuals and triads. These results could be explained by the odd-vs. even-numbered group dynamics (Asch, 1951; Wittenbaum et al., 1996; Hastie and Kameda, 2005; Menon and Phillips, 2011; Harada, 2021b) and the knowledge diversity inherent in larger groups (Hülsheger et al., 2009). The results could be consistently interpreted in the context of these models. Third, though future studies should examine the robustness of the results, the Q learning framework as measures for learning properties of both individuals and groups should remain valid, regardless of the results. A standard method for evaluating group dynamics and related learning draw upon questionnaire surveys. However, this method suffers from the subjectivity of respondents. The Q learning computational methods are based on observed, objective data on individual and collective decision-making. Hence, the computational approach could at least complement, if not substitute, the traditional questionnaire methods on group dynamics.

Some recent studies on the computational approach to decision-making under uncertainty using multi-armed bandits have shifted attention from single-agent to multi-agent policies with rigorous stochastic setting and communication design across agents (Kolla et al., 2016; Shahrampour et al., 2017; Landgren et al., 2018; Wei and Srivastava, 2018; Landgren et al., 2021). However, these studies primarily rely on mathematical models and numerical simulation without resorting to human behavior. While this study did not specify communication routes across group members, it differs as the results were derived from human experiments.

Notably, our results were consistent with the representational change theory in insight problem-solving (Ohlsson, 1992; Knöblich et al., 1999). According to this theory, insight problems generate impasses due to the construction of an erroneous problem space. The impasses are efficiently resolved by a representational change in problem spaces through constraint relaxation, which is enabled by the interplay between conscious and unconscious processes (Smith and Kounios, 1996; Topolinski and Reber, 2010). Risk-taking and exploration might induce this representational change because they facilitate new challenges. This is shown in the result that either risk-taking or exploration in individuals and triads was positively related to divergent thinking. Although neither exploration nor risk-taking was significant in dyads, constraint relaxation could be inferred to be initiated by learning incoherence caused by group dynamics in dyads.

Our computational approach to divergent thinking and learning highlighted the underlying learning properties not explicitly modeled in the representational change literature. Although the Q learning model was only applied to the TAB in this study, this framework could be used in divergent thinking. Under this framework, the representational change is caused by shifting to non-optimal existing or new choices. By definition, exploration leads to random (thus usually non-optimal) choices, and risk-taking encourages shifts to new options. Thus, it could be inferred that representational change is facilitated by either exploration or risk-taking, increasing Q values of non-optimal options.

In either case, the representational change is facilitated through increasing Q values of non-optimal options. This study’s results revealed the fact that at least either of the two is required for high divergent thinking. Indeed, the relative contribution to divergent thinking could not be precisely evaluated without explicitly modeling these relevant parameters. This result also accounts for the advantage of the computational approach to divergent thinking.

The Q learning framework has been supported by several empirical evidence, including neural signals in various cortical and subcortical structures that behaved as predicted (Schultz et al., 1997; Glimcher and Rustichini, 2004; Hikosaka et al., 2006; Rangel et al., 2008). However, regarding divergent thinking, several neuroimaging studies indicated that any clear neuroanatomical localization of creative processes failed to exist (Dietrich and Kanso, 2010; Mihov et al., 2010). Instead, creative processes have been associated with many different cognitive and affective processes (Kizilirmak et al., 2016; Shen et al., 2018). Our results highlighted the importance of balancing both boldness (risk-taking, exploration, learning incoherence) and prudence (risk-aversion, exploitation, loss aversion) in individuals and triads. One of our future challenges is to integrate the diverse neural processes associated with creativity with these balancing forces in a simple Q-learning framework.

## Conclusion

This study examined the effects of risk-taking and learning properties on divergent thinking by taking a computational approach. First, we adopted a simple Q learning model to estimate risk attitudes and learning parameters in the TAB. Then, the statistical relationship between these parameters and divergent thinking scores was evaluated. The results indicated that these effects differed across individuals, dyads, and triads. On the one hand, in dyads, it was inferred that learning incoherence emerged, enabling divergence in ideas across group members without relying on risk attitudes and learning parameters. On the other hand, risk attitudes and the inverse temperature as a ratio between exploitation and exploration were significant but with contrasting effects in both individuals and triads. For individuals, risk-taking and exploitation played a critical role in divergent thinking. However, risk aversion and exploration were significantly related to divergent thinking for triads. This difference could be explained by increased group size leading to greater knowledge diversity. Thus, triads with more knowledge diversity required risk aversion. However, individuals, by nature less diverse, needed to take risks.

Furthermore, the effects of loss aversion showed contrasting results between individuals and dyads. For individuals, loss aversion was positively related to divergent thinking whereas its effects were negative in dyads.

Despite these contrasting effects of risk attitudes, that are the inverse temperature and loss aversion, the results also indicated the importance of balance between risk attitudes, inverse temperature, and loss aversion. For example, high divergent thinking individuals required risk-taking, but this was complemented by exploitation and loss aversion. Similarly, high divergent thinking triads relied on risk aversion, complemented by exploration. High divergent thinking dyads necessitated low levels of loss aversion due to their learning incoherence. Thus, balancing risk, exploitation/exploration and loss aversion seems critical in enhancing divergent thinking. In other words, divergent thinking requires both boldness (risk-taking, exploration, learning incoherence) and prudence (risk-aversion, exploitation, loss aversion) in individuals and triads. Regarding dyads, since learning incoherence emerges, these balancing forces might not play a critical role in divergent thinking. In other words, balancing forces are required only if learning coherence takes place.

Without a doubt, exploration of the determinants of creativity in group dynamics could constitute one of the important research topics in creativity literature. While previous studies in group dynamics tended to resort to questionnaire surveys, the computational approach proposed in this study could be a promising alternative approach to group dynamics and creativity. This study is the first attempt to apply the computational approach to creativity under group dynamics to the best of our knowledge. We hope this approach will be widely used in future studies to elucidate underlying cognitive and psychological mechanisms.

## Data availability statement

The raw data supporting the conclusions of this article will be made available by the authors, without undue reservation.

## Ethics statement

The studies involving human participants were reviewed and approved by The Ethics Committee at the Graduate School of Business Administration, Kobe University. The patients/participants provided their written informed consent to participate in this study.

## Author contributions

The author confirms being the sole contributor of this work and has approved it for publication.

## Funding

This work was supported by JSPS KAKENHI under Grant (Number 26380506).

## Conflict of interest

The author declares that the research was conducted in the absence of any commercial or financial relationships that could be construed as a potential conflict of interest.

## Publisher’s note

All claims expressed in this article are solely those of the authors and do not necessarily represent those of their affiliated organizations, or those of the publisher, the editors and the reviewers. Any product that may be evaluated in this article, or claim that may be made by its manufacturer, is not guaranteed or endorsed by the publisher.
